# Learning Curve for Endovascular Treatment of Anterior Circulation Large Vessel Occlusion at a Single Center

**DOI:** 10.3389/fneur.2020.587409

**Published:** 2021-01-14

**Authors:** Qiankun Cai, Yuyou Zhu, Xianjun Huang, Lulu Xiao, Mengmeng Gu, Peng Wang, Chao Zhang, Jixing Chen, Wei Hu, Guoping Wang, Wen Sun

**Affiliations:** ^1^Department of Neurology, Second Affiliated Hospital, Fujian Medical University, Quanzhou, China; ^2^Department of Neurology, Stroke Center, The First Affiliated Hospital of University of Science and Technology of China, Hefei, China; ^3^Department of Neurology, Yijishan Hospital of Wannan Medical College, Wuhu, China; ^4^Department of Neurology, Jinling Hospital, Medical School of Nanjing University, Nanjing, China; ^5^Department of Neurology, Nanjing First Hospital, Nanjing Medical University, Nanjing, China; ^6^Department of Radiology, Stroke Center, The First Affiliated Hospital of University of Science and Technology of China, Hefei, China

**Keywords:** learning curve, endovascular treatment, large vessel occlusion, risk adjusted cumulative sum, stroke

## Abstract

**Background and purpose:** Data concerning the learning curve for endovascular treatment (EVT) of anterior circulation large vessel occlusion are scarce. This study aimed to investigate the relationship between operator experience and the outcome of EVT and to further identify the number of cases needed to acquire the ability to perform successful reperfusion.

**Materials and methods:** Four hundred and thirty-four patients who underwent EVT by seven operators at a single center from January 2016 to September 2019 were enrolled. Procedural experience was defined by the number of cases performed by each operator. Multivariable backward regression analyses were used to investigate the association between procedural experience and functional independence (defined as a modified Rankin Scale score of 0–2), 90-days mortality, successful reperfusion (defined as a modified Thrombolysis in Cerebral Infarction score of 2b-3), and puncture-to-reperfusion time after adjusting for covariates. A risk-adjusted cumulative sum (RA-CUSUM) chart was utilized to identify the number of caseloads needed to overcome the learning curve effect.

**Results:** Procedural experience was independently associated with functional independence, 90-days mortality, successful reperfusion, and puncture-to-reperfusion time reduction (per 10-case increment: OR 1.219, 95% CI: 1.079–1.383, *P* < 0.001; OR 0.847, 95% CI: 0.738–0.968, *P* = 0.016; OR 1.553, 95% CI: 1.332–1.830, *P* < 0.001 and β 8.087 min, 95% CI: 6.184–9.991, *P* < 0.001, respectively). The RA-CUSUM chart indicated that at least 29 cases were required to overcome the learning curve effect.

**Conclusions:** There was a dose-response relationship between operator case volume and clinical outcome, procedure time, and successful reperfusion. The experience needed for successful EVT was at least 29 cases.

## Introduction

Endovascular treatment (EVT) became the standard therapy for anterior circulation large vessel occlusion stroke (LVOS) after landmark clinical trials were published in 2015 (1–5). Previous studies have indicated that a higher procedure volume is associated with a higher proportion of reperfusion and favorable clinical outcomes (6–8). However, in the real world, it is difficult for high-volume hospitals to provide access to this time-dependent therapy to every eligible patient because of the geographic maldistribution of neurointerventionalists. An increasing prevalence of EVT performed by less-experienced operators has been seen in hospitals in recent years (9). To ensure adequate procedural quality, describing the learning curve for EVT and establishing a caseload threshold for operators are urgently needed. Therefore, based on a prospective database, we investigated the relationship between operator experience and the outcome of EVT and further identified the specific number of cases needed to acquire technical success.

## Materials and Methods

### Patient Selection

We retrospectively analyzed the clinical data of all consecutive patients with LVOS who underwent EVT by seven neurologists at a single center from January 2016 to September 2019. Each neurologist was accredited to perform carotid and intracranial stenting by the national accrediting institution before 2013. We performed the first EVT case in the year 2016 in our center; however, before that time, all seven neurologists had completed a 1-year additional fellowship of endovascular mechanical thrombectomy. In general, patients with acute ischemic stroke were enrolled in this study if they fulfilled the following criteria: (1) age ≥18 years, (2) anterior circulation vessel occlusion confirmed by radiographic imaging, (3) baseline National Institutes of Health Stroke Scale (NIHSS) score ≥6, (4) premorbid modified Rankin Scale (mRS) score of <2, and (5) time from symptom onset to puncture time <24 h. The study excluded patients treated with posterior circulation LVOS.

### Procedure for Endovascular Treatment

The EVT procedures included the use of stent retrievers, aspiration, angioplasty, stenting, or combinations of these approaches. If reocclusion occurred after thrombectomy, the rescue therapy, including angioplasty, stenting, intra-arterial thrombolysis, and glycoprotein IIb/IIIa inhibitor infusion, were allowed. The choice of treatment option, material used for EVT, and type of anesthesia was left to the discretion of the operators. In our institution, the Solitaire stent (Covidien), was the only stent retriever available. Furthermore, balloon-guide catheters were unavailable. The blood pressure after the procedurals was kept <130/80 mmHg if recanalization was achieved; otherwise, the blood pressure was controlled under 180/100 mmHg. All patients were monitored over at least a 24-h period after the procedures.

All procedures were performed during and off hours with the same staff (i.e., including a primary operator, an assistant at the operation table, an experienced nurse, and a radiology technician).

### Data Collection and Outcome Evaluation

Fourteen patient-related, seven treatment-related, and two operator-related. Patient-related variables included age, sex, NIHSS score, hypertension, diabetes mellitus, dyslipidemia, atrial fibrillation, smoking, stroke cause (large artery atherosclerosis, cardioembolism, and dissection/undetermined cause), baseline Alberta Stroke Program Early CT score (ASPECTS) (10) affected hemisphere, occlusion site, including intracranial internal carotid artery (ICA) or M1/proximal M2 segment of the middle cerebral artery (MCA) or anterior cerebral artery occlusion, tandem lesion (defined as carotid stenosis ≥90% or complete occlusion confirmed by radiographic imaging) and collateral score with a 4-point scale (0, no filling of the occluded territory; 1, >0% and ≤ 50% filling of the occluded territory; 2, >50% and <100% filling of the occluded territory; 3, 100% filling of the occluded territory) (11) and was dichotomized as poor (0–1) or good collaterals (2–3).

Treatment-related variables included onset-to-puncture time (OPT), door-to-puncture time (DPT), intravenous thrombolysis, intracranial occlusion treatment option (stent retrieval, angioplasty/intracranial stent, aspiration), type of anesthesia, cervical carotid artery stenting, and additional rescue therapy.

Operator-related variables included operators and procedural experience. Procedural experience was defined as the number of cases performed by each individual operator (i.e., operator's 1st, 2nd, 3rd, … or nth case). In the case of two operators with different levels of case experience performing a procedure together, the case was categorized based on the number of cases of the more experienced operator. Procedural experience was graded as three levels (1–20 cases, 21–40 cases, and >40 cases) for univariate analysis.

The EVT prognostic outcome was functional independence (defined as mRS score of 0–2 at 90 days) and mortality within 90 days.

The primary EVT technical outcome was successful reperfusion (SR), which was defined as the achievement of a modified Thrombolysis in Cerebral Infarction score (mTICI) of 2b-3, as determined according to the final angiogram after the procedure. The secondary technical outcome was puncture-to-reperfusion time (PRT), which was defined as the time interval from puncture to final SR or to abortion of the procedure if SR was not achieved.

The safety outcome was symptomatic intracerebral hemorrhage (SICH), which was defined as any parenchymal hematoma, subarachnoid hemorrhage, or intraventricular hemorrhage associated with a worsening of the NIHSS score by ≥4 points within 36 h after the procedure in this study.

All radiological imaging data, including occlusion sites, ASPECTS, type of hemorrhagic transformation, collateral score, and mTICI post-procedure were sent to the core laboratory in Jingling Hospital and were evaluated in a blind manner by two physicians (LL, X and P, W). Any disagreement was resolved by consensus.

### Statistical Analysis

Continuous variables are presented as the median (interquartile range, IQR), and categorical variables are presented as the frequency (percentage). Univariate analysis was compared with Kruskal-Wallis test or Mann-Whitney *U* test for continuous variables and chi-square or Fisher's exact test for categorical variables. Covariates associated with PRT were analyzed using bivariate correlation analysis. Multivariable backward regression analyses were used to investigate the association between procedural experience (as a continuous variable) and functional independence (logistic), 90-days mortality (logistic), SR (logistic), and PRT (linear) adjusting covariates with *P* < 0.10 in univariate analyses. Relationships between procedural experience and outcomes were plotted as curves based on multivariable backward regression models with the mean values of other significant covariates.

To identify the specific number of cases needed to acquire SR, the risk-adjusted cumulative sum chart (RA-CUSUM) was plotted; another logistic backward model was constructed for SR itself, regardless of procedural experience, only using covariates with *P* < 0.10 in univariate analyses. The details of the RA-CUSUM approach are provided in the online-only Supplemental Material, and this approach has been described in a previous study (12). In brief, the RA-CUSUM chart demonstrated the difference between cumulative predicted and observed events, with the X-axis representing the number of cases the operator had performed. In the inexperienced phase, the cumulative predicted SR was greater than the observed SR, as demonstrated by the ascending graph. In the plateau phase, the cumulative predicted SR was comparable to the observed SR, as shown by the leveling off. After achieving enough experience, the cumulative predicted SR was less than the observed SR, as displayed by the descending graph. Therefore, there ought to be a turning point, which could represent the specific number of cases needed to require SR.

Missing data occurred at a rate of <5% for all covariates, and it was imputed with the median for continuous variables and with the mode for categorical variables. For all analyses, a two-tailed value of *P* ≤ 0.05 was considered significant. All statistical analyses were performed using SAS version 9.4 (SAS Institute Inc., Cary, North Carolina).

### Ethics Approval

This study was approved by the Institutional Review Boards of our center. Patient consent was waived due to the retrospective nature of the study.

## Results

A total of 470 consecutive patients who underwent EVT by seven operators were enrolled from January 2016 to September 2019. Of these, 36 patients were excluded because of posterior circulation occlusion. The remaining 434 patients all underwent 90-days follow-up and were entered for the data analysis. Of these, 150 (34.6%) patients were female, 82 (18.9%) patients were diagnosed with tandem lesions, and 377 (86.9%) patients were treated by stent retrieval. The median age, baseline NIHSS score, ASPECTS, and OPT were 67 years (IQR, 57–74), 17 (IQR, 13–21), 9 (IQR, 8–10), and 285 min (IQR, 225 min−363 min), respectively. The numbers of procedures performed by each of the seven operators were 61, 54, 56, 54, 58, 79, and 72.

Of the enrolled patients, 348 (80.2%) patients achieved SR, 51 (11.8%) had SICH, and in 10 (2.3%), the guidewire failed to cross the occlusion site. There were eight patients with missing PRT data, and the median PRT in the remaining patients was 73 min (IQR, 47 min-105 min). There were 193 (44.5%) patients who achieved 90-days functional independence, and 100 (23.0%) patients died.

Patient- and treatment-related variables stratified according to the three levels of procedural experience are compared in Table 1. There was no significant difference in the other variables, except for baseline NIHSS, dyslipidemia, OPT, and type of anesthesia among the three levels.

**Table 1:** Patient-related and treatment-related variables according to procedural experience categories.

**Variables**	**1–20 cases (*N* = 140)**	**21–40 cases (*N* = 140)**	**>40 cases (*N* = 154)**	***P***
Age, median (IQR), y	68 (56–75)	65 (55–73)	68 (58–74)	0.561
Male, *n* (%)	96 (68.6)	85 (60.7)	103 (66.9)	0.345
NIHSS, median (IQR)	18 (15–22)	18 (14–21)	16 (12–19)	<0.001
Hypertension, *n* (%)	94 (67.1)	92 (65.7)	94 (61.0)	0.516
Diabetes mellitus, *n* (%)	40 (28.6)	29 (20.7)	32 (20.8)	0.197
Dyslipidemia, *n* (%)	32 (22.9)	43 (30.7)	28 (18.2)	0.040
Atrial fibrillation, *n* (%)	62 (44.3)	55 (39.3)	52 (33.8)	0.181
Current Smoking, *n* (%)	39 (27.9)	48 (34.3)	52 (33.8)	0.436
Stroke causes, *n* (%)				0.539
Cardioembolism	66 (47.1)	64 (45.7)	68 (44.2)	
Large artery atherosclerosis	57 (40.7)	62 (44.3)	75 (48.7)	
Other	17 (12.1)	14 (10.0)	11 (7.1)	
ASPECTS, median (IQR)	9 (7–10)	9 (8–10)	9 (8–10)	0.648
Occlusion site, *n* (%)				0.378
MCA M1	65 (46.4)	79 (56.4)	90 (58.4)	
Internal carotid artery	59 (42.1)	47 (33.6)	51 (33.1)	
MCA M2	14 (10.0)	13 (9.3)	10 (6.5)	
Anterior cerebral artery	2 (1.4)	1 (0.7)	3 (1.9)	
Tandem lesion, *n* (%)	32 (22.9)	24 (17.1)	26 (16.9)	0.346
Collateral, *n* (%)				0.192
Poor (0–1)	73 (52.1)	65 (46.4)	64 (41.6)	
Good (2–3)	67 (47.9)	75 (53.6)	90 (58.4)	
Affected hemisphere, *n* (%)				0.096
Left	88 (62.9)	71(50.7)	82 (53.2)	
Right	52 (37.1)	69 (49.3)	72 (46.8)	
OPT, median (IQR), min	270 (220–339)	279 (216–359)	305 (240–392)	0.011
DPT, median (IQR), min	113 (90–130)	107 (87–139)	114 (82–150)	0.881
IV Thrombolysis, *n* (%)	44 (31.4)	44 (31.4)	40 (26.0)	0.490
Retriever stent, *n* (%)	118 (84.3)	126 (90.0)	133 (86.4)	0.358
Angioplasty/stent, *n* (%)	9 (6.4)	10 (7.1)	11 (7.1)	0.963
Aspiration, *n* (%)	5 (3.6)	2 (1.4)	10 (6.5)	0.079
Type of anesthesia, *n* (%)				0.012
General anesthesia	3 (2.1)	11 (7.9)	17 (11.0)	
Local anesthesia	137 (97.9)	129 (92.1)	137 (89.0)	
Carotid stent, *n* (%)	10 (7.1)	8 (5.7)	9 (5.8)	0.859
Rescue therapy, *n* (%)	57 (40.7)	53 (37.9)	77 (50.0)	0.087

*NIHSS, National Institutes of Health Stroke Scale; ASPECTS, Alberta Stroke Program Early CT score; MCA, middle cerebral artery; OPT, onset-to-puncture time; DPT, door-to-puncture time; IV, intravenous*.

**Figure 1:**
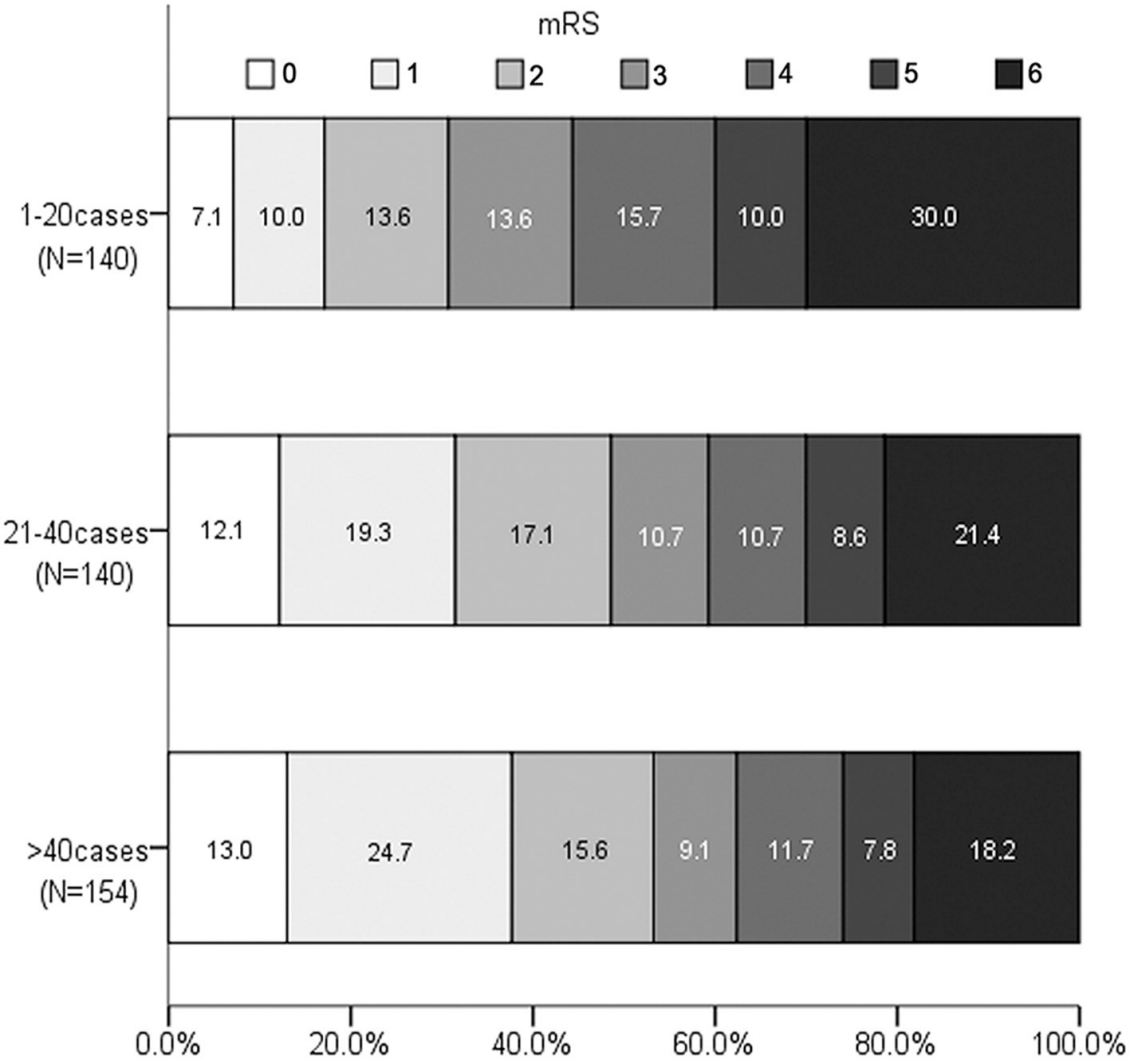
Distribution of Modified Rankin Scale score at 90 days according to experience categories.

For the outcome variables, the rate of SR and functional independence gradually increased across the three levels of procedural experience. The median of PRT, mRS at 90 days, and the rate of mortality gradually decreased. The distribution of mRS is shown in Figure 1. However, the proportion of SICH was not significantly different with the accumulation of experience (Table 2).

**Table 2:** Technical, clinical, and safety outcomes according to procedural experience categories.

**Outcome**	**1–20 cases (*N* = 140)**	**21–40 cases (*N* = 140)**	**>40 cases (*N* = 154)**	***P***
Functional independence, *n* (%)	43 (30.7)	68 (48.6)	82 (53.2)	<0.001
90-days mortality, *n* (%)	42 (30.0)	30 (21.4)	28 (18.2)	0.048
PRT, median (IQR), min	92 (58–139)^†^	83 (53–107)^‡^	52 (43–78)^§^	<0.001
Successful reperfusion, *n* (%)	89 (63.6)	119 (85.0)	140 (90.9)	<0.001
SICH, *n* (%)	19 (13.6)	16 (11.4)	16 (10.4)	0.692
mRS, median (IQR)	4 (2–6)	3 (1–5)	2 (1–5)	<0.001

*PRT, puncture-to-reperfusion time; SICH, symptomatic intracerebral hemorrhage; mRS, modified Rankin scale*.

† *There were six patients with missing data*.

‡ *There was one patient with missing data*.

§ *There was one patient with missing data*.

Moreover, the rates of SR, functional independence, mortality, SICH, and the median of PRT and mRS were not significantly different among the seven operators (see Supplementary Table 1).

### The Relationship Between Procedural Experience and Functional Independence and 90-Days Mortality

**Figure 2:**
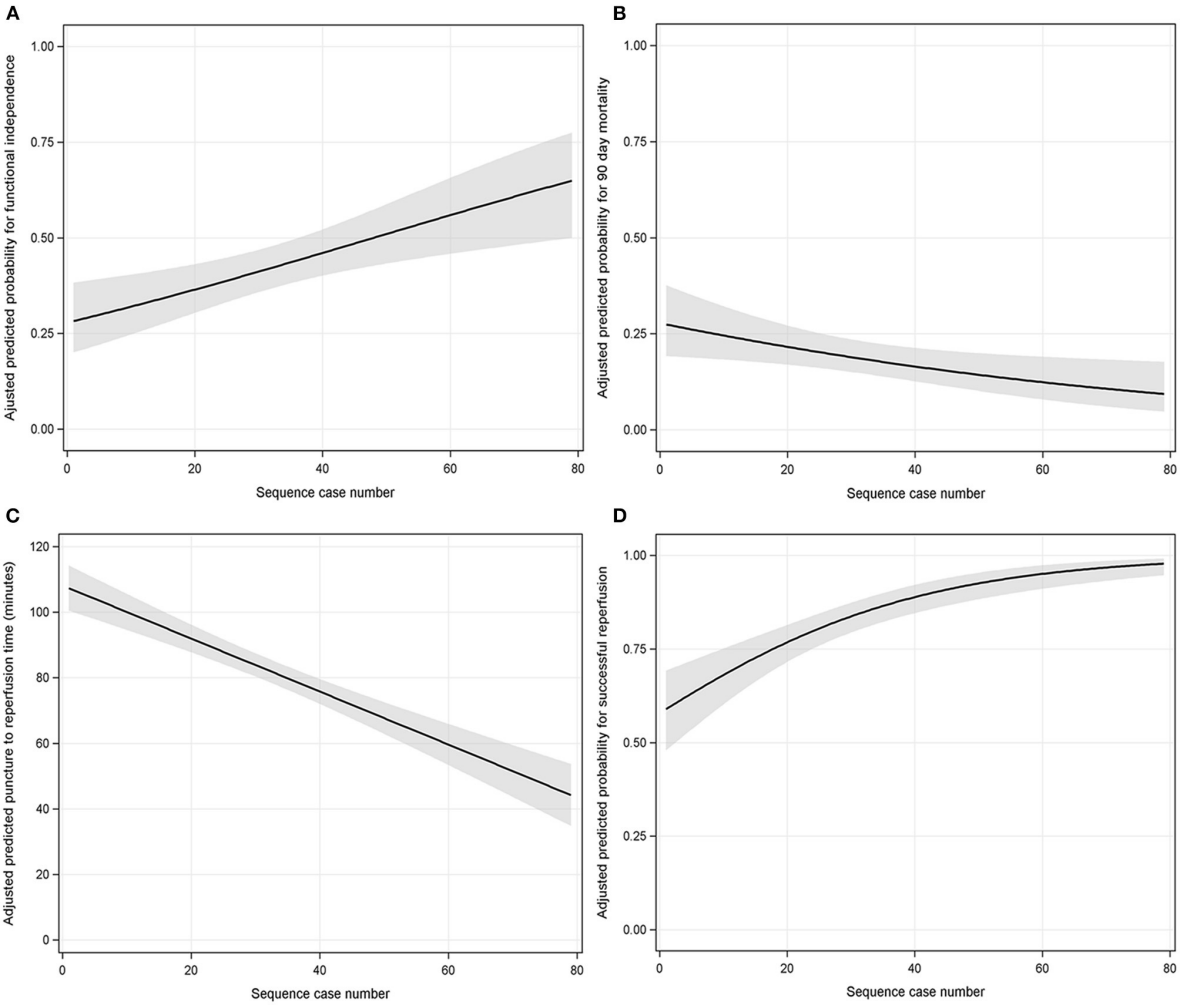
The association between procedural experience and outcomes (**A** = functional independence; **B** = 90-days mortality; **C** = puncture-to-reperfusion time; **D** = successful reperfusion). The black line with shaded bands represents the adjusted predicted curve with 95% confidence interval.

After adjusting for covariates (see Supplementary Table 2), multivariable logistic backward regression showed that procedural experience was independently associated with functional independence and 90-days mortality reduction (per 10-case increment: OR 1.219, 95% CI: 1.079–1.383, *P* < 0.001 and OR 0.847, 95% CI: 0.738–0.968, *P* = 0.016, both see Supplementary Table 4). The adjusted predicted probability for functional independence and 90-days mortality with procedural experience are depicted in Figures 2A,B, respectively.

### The Association Between Procedural Experience and PRT and SR

Similarly, adjusting for covariates (see Supplementary Table 3), procedural experience was independently associated with a reduction in PRT (per 10-case increment: β 8.087 min, 95% CI: 6.184–9.991, *P* < 0.001, see Supplementary Table 6). The predicted PRT with procedural experience is depicted in Figure 2C.

Multivariable logistic backward regression-adjusted covariates (see Supplementary Table 2) demonstrated that procedural experience was independently associated with SR (per 10-case increment: OR 1.553, 95% CI: 1.332–1.830, *P* < 0.001, see Supplementary Table 4). The predicted probability for SR with procedural experience is depicted in Figure 2D. We could visually observe that there was a clear ceiling effect for SR.

### The Relationship Between Procedural Experience and SICH

Procedural experience showed no significant correlation with SICH (*r* = −0.072, *P* = 0.134). In the multivariable model, current smoking and collateral circulation were independently associated with SICH (see Supplementary Table 5).

### Risk-Adjusted Cumulative Sum Chart

**Figure 3:**
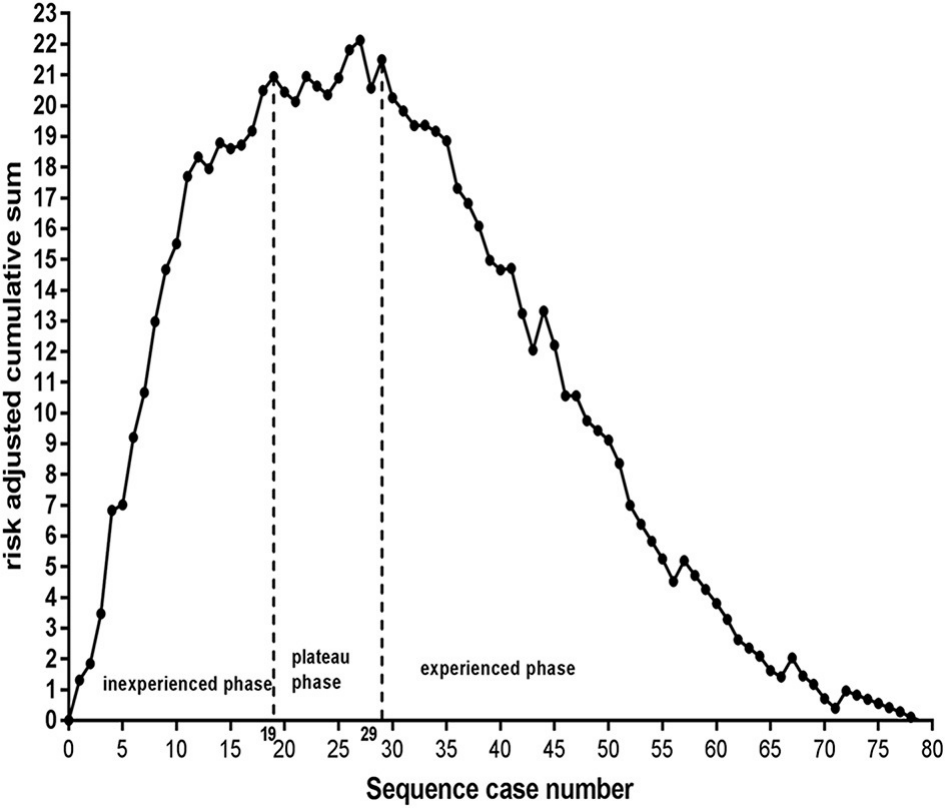
Inexperience phase: from the 1st to the 19th case, plateaus phase: from the 19th to 29th case, experience phase: after the 29th case. The threshold required to achieve successful reperfusion is 29 cases.

To accurately measure the threshold of EVT, the predicted probability of SR itself, regardless of procedural experience, was calculated with another logistic backward model for the RA-CUSUM chart (see Supplementary Table 6). The RA-CUSUM chart demonstrated that the predicted SR was greater than the observed SR before the 19th consecutive case (ascending graph) and that the predicted SR was comparable to the observed SR from the 19th to the 29th consecutive case (leveling off). However, the predicted SR was less than the observed SR after the 29th case (descending graph), as shown in Figure 3. This finding suggests that the threshold of EVT required to achieve SR is 29 cases.

After experience with 29 cases, the comparison of outcomes between our study and the published data from Highly Effective Reperfusion evaluated in Multiple Endovascular Stroke Trials (HERMES) collaboration (13), is shown in Table 3. The rates of SR, functional independence, and SICH in our study were significantly higher than those of the HERMES collaboration. However, the proportion of 90-days mortality was not significantly different.

**Table 3:** Comparison between the results of our study after experience with 29 cases and the published data from the HERMES collaboration.

	**Our study (*N* = 231)**	**HERMES (*N* = 634)**	***P***
Successful reperfusion, % (n/N)	90.5 (209/231)	70.5 (402/570)	<0.001
Functional independence, % (n/N)	57.3 (124/231)	46.0 (291/633)	0.045
90-days mortality, % (n/N)	18.6 (43/231)	15.3 (97/633)	0.245
SICH, % (n/N)	9.1 (21/231)	4.4 (28/634)	0.009

*SICH, symptomatic intracerebral hemorrhage*.

**Figure 4:**
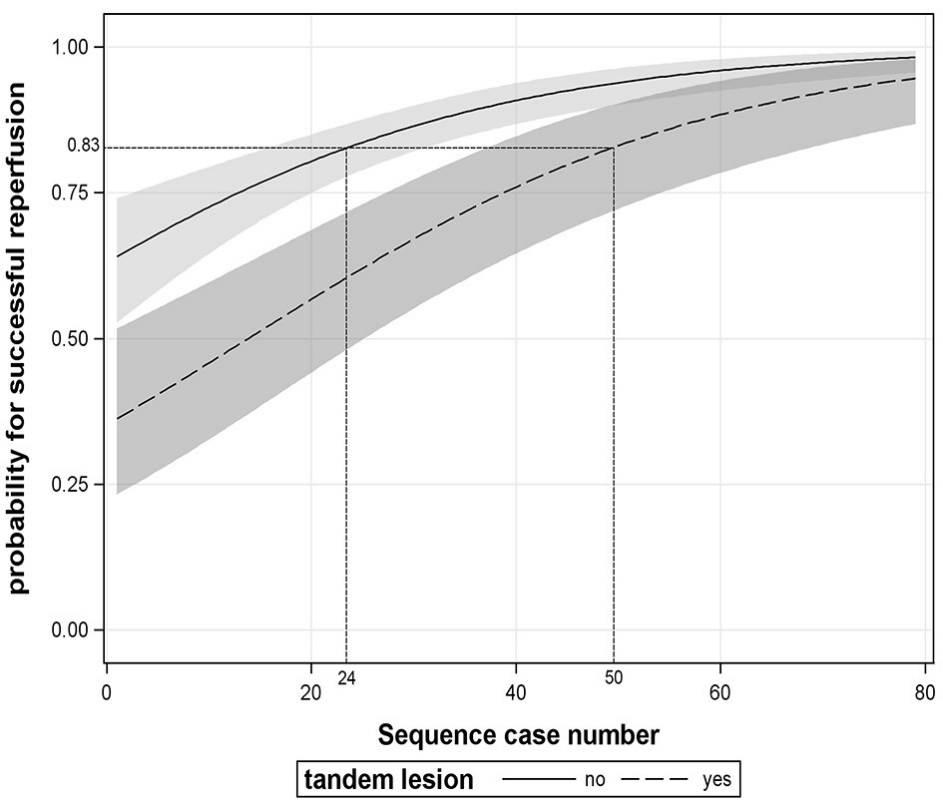
The association between procedural experience and successful reperfusion stratified by tandem lesion. The black line and dotted line with shaded bands represent the adjusted predicted curves with 95% confidence interval.

In addition, we found that patients with tandem lesions had a lower proportion of SR than those with isolated intracranial artery occlusion (OR 0.320, 95% CI: 0.177–0.578, *P* < 0.001, see Supplementary Table 4). The average predicted probability of SR in the 29th case was 83% according to Figure 2D. The operators needed to complete at least 24 cases without tandem lesions to obtain an equivalent predicted probability of SR; however, for patients with tandem lesions, the number of procedures required substantially increased to 50, as shown in Figure 4.

## Discussion

Our study demonstrated that operator experience significantly increased the likelihood of favorable clinical outcomes and reduced the likelihood of mortality. This finding is supported by a recent large-sample study (8). This phenomenon is mainly attributed to the shorter procedural time needed and the greater SR achieved with accumulated experience.

It is well-known that the benefit of EVT is highly time-dependent (14). Recently, a multicenter study indicated that shorter procedure time led to higher rates of functional independence and lower rates of 90-days mortality (15). Our study showed a dose-response relationship between the PRT and the operator's experience although the magnitude of PRT reduction was small, an 8 min reduction per 10-case increment. However, the effect was obvious with the accumulation of experience; the median PRT reduced from 92 min at the 1–20 cases category to 52 min at the >40 cases category.

Previous studies reported that higher procedure volume is associated with a higher proportion of SR (6–8), and our study found a similar phenomenon. In addition, we observed that the predicted rate of SR quickly reached a plateau. The final status of reperfusion represent the strongest predictors of prognostic outcome in patients who undergo EVT (16). Unlike the ceiling effect observed with SR, the predicted rate of functional independence continuously improved as experience increased. This suggested that the goal of SR was easily achieved with procedural experience. However, functional independence might rely more on patient characteristics (e.g., baseline NIHSS score was significantly lower at the >40 cases category in our study), not just SR.

Because of a ceiling effect on SR, there should be a threshold for procedural experience. In addition to operator skill, the learning curve depends on other factors, including procedure complexity and patient characteristics. Therefore, describing the learning curve should adjust for case mixing. The RA-CUSUM chart exactly solves this problem. We plotted the RA-CUSUM chart to identify operators who needed 29 cases to overcome the learning curve effect. In our study, after 29 cases, the rates of SR, functional independence, and 90-days mortality were 90.5, 57.3, and 18.6%, respectively. The results are numerically not inferior to those of the HERMES collaboration (13). As we all know, these trials were exclusively conducted by experienced operators in high-volume centers. Therefore, we suggest that operators can obtain beneficial effects after experience with 29 cumulative cases of EVT based on the pooled results of five trials.

It is well-known that SICH occurred in 11.8% of patients in our study, which is significantly more than that reported by the HERMES collaboration (4.4%) (13). However, it is consistent with previously published real-world data from China. The Endovascular Treatment for Acute Anterior Circulation Ischemic Stroke Registry reported 16.0% incidence of SICH after EVT (17). Our study may reflect real-world practice, as we enrolled more patients with disadvantageous treatment conditions. For example, compared to 15% of patients in the HERMES collaboration, which used the same method to assess collaterals, 46.5% (202/434) of the patients enrolled in our study had poor collaterals compared to 15% patient in HERMES collaboration (18). Moreover, the rate of SICH did not significantly decrease as operator experience increased in our study. This phenomenon may partly be attributable to the insufficient case volume. According to a recent study, when the operator case volume was limited within 150 cases, no significant correlation between case volume and SICH could be found (*r* = −0.05, *P* = 0.178) (8).

In addition, in our present study, it is not surprising that patients with tandem occlusions had a lower SR rate than those with isolated intracranial occlusion because the additional complicated manipulation involved in tandem occlusion treatment inevitably results in a longer learning process. This phenomenon was also noticed in a recently published study (19). However, in contrast to our expectations, the number of EVT procedures needed to treat, for patients with tandem lesions, was more than twice as much as the number needed for patients with isolated intracranial occlusions (50 vs. 24) in order to reach the target level of SR. There have been no relevant data to date that support our results. Therefore, further confirmation is needed.

Our study has some limitations. First, the learning curve in the present study is representative of only the classic approaches and devices, not the new ones (e.g., balloon guide catheter) that have been developed in recent years (20, 21). Second, we excluded patients with vertebral-basilar LVOS because the methods for evaluating ASPECTS and collateral score in posterior circulation are different from anterior circulation (10, 11, 22, 23). Despite the proportion of posterior circulation-only accounting for 7.7% (36/470) of all LVOS treated by EVT in our study, this may lead to underestimating the pitfalls of the learning curve because of the similarity of EVT for anterior and posterior circulation. Third, our results describe the performance of seven operators in a single center; further validation is needed from multicenter data.

## Conclusions

Our data suggested that there was a dose-response relationship between operator case volume and clinical outcome, procedure time reduction, and SR. Moreover, the experience level needed to perform EVT successfully was at least 29 cases in this study.

## Data Availability Statement

The raw data supporting the conclusions of this article will be made available by the authors, without undue reservation.

## Ethics Statement

The studies involving human participants were reviewed and approved by the First Affiliated Hospital of University of Science and Technology of China. Written informed consent for participation was not required for this study in accordance with the national legislation and the institutional requirements.

## Author Contributions

WS and GW conceived, designed, and supervised the study. XH, LX, MG, CZ, WH, and JC acquired the data. QC and YZ analyzed and interpreted the data, provided statistical analysis, responsible for the integrity of the data, accuracy of the data analysis, and drafted the manuscript. WS, GW, and WH critically revised the manuscript for important intellectual content. All authors contributed to the article and approved the submitted version.

## Conflict of Interest

The authors declare that the research was conducted in the absence of any commercial or financial relationships that could be construed as a potential conflict of interest.

## References

[1] Saver JL Goyal M Bonafe A Diener HC Levy EI Pereira VM. Stent-retriever thrombectomy after intravenous t-PA vs. t-PA alone in stroke. N Engl J Med. (2015) 372:2285–95. 10.1056/NEJMoa1415061 25882376

[2] Campbell BC Mitchell PJ Kleinig TJ Dewey HM Churilov L Yassi N. Endovascular therapy for ischemic stroke with perfusion-imaging selection. N Engl J Med. (2015) 372:1009–18. 10.1056/NEJMoa1414792 25671797

[3] Goyal M Demchuk AM Menon BK Eesa M Rempel JL Thornton J. Randomized assessment of rapid endovascular treatment of ischemic stroke. N Engl J Med. (2015) 372:1019–30. 10.1056/NEJMoa1414905 25671798

[4] Berkhemer OA Fransen PS Beumer D van den Berg LA Lingsma HF Yoo AJ. A randomized trial of intraarterial treatment for acute ischemic stroke. N Engl J Med. (2015) 372:11–20. 10.1056/NEJMoa1411587 25517348

[5] Jovin TG Chamorro A Cobo E de Miquel MA Molina CA Rovira A. Thrombectomy within 8 h after symptom onset in ischemic stroke. N Engl J Med. (2015) 372:2296–306. 10.1056/NEJMoa1503780 25882510

[6] Gupta R Horev A Nguyen T Gandhi D Wisco D Glenn BA. Higher volume endovascular stroke centers have faster times to treatment, higher reperfusion rates and higher rates of good clinical outcomes. J Neurointerv Surg. (2013) 5:294–7. 10.1136/neurintsurg-2011-010245 22581925

[7] El Nawar R Lapergue B Piotin M Gory B Blanc R Consoli A. Higher annual operator volume is associated with better reperfusion rates in stroke patients treated by mechanical thrombectomy: the ETIS registry. JACC Cardiovasc Interv. (2019) 12:385–91. 10.26226/morressier.5ab8f561d462b8029238cec3 30784645

[8] Kim BM Baek JH Heo JH Kim DJ Nam HS Kim YD. Effect of cumulative case volume on procedural and clinical outcomes in endovascular thrombectomy. Stroke. (2019) 50:1178–83. 10.1161/STROKEAHA.119.024986 30943886

[9] Saber H Navi BB Grotta JC Kamel H Bambhroliya A Vahidy FS. Real-World treatment trends in endovascular stroke therapy. Stroke. (2019) 50:683–89. 10.1161/STROKEAHA.118.023967 30726185 PMC6407696

[10] Barber PA Demchuk AM Zhang J Buchan AM. Validity and reliability of a quantitative computed tomography score in predicting outcome of hyperacute stroke before thrombolytic therapy. ASPECTS Study Group. Alberta Stroke Programme Early CT Score. Lancet. (2000) 355:1670–4. 10.1016/S0140-6736(00)02237-6 10905241

[11] Berkhemer OA Jansen IG Beumer D Fransen PS van den Berg LA Yoo AJ. Collateral status on baseline computed tomographic angiography and intra-arterial treatment effect in patients with proximal anterior circulation stroke. Stroke. (2016) 47:768–76. 10.1161/STROKEAHA.115.011788 26903582

[12] Cai Q Li Y Xu G Sun W Xiong Y Bao Y. Learning curve for intracranial angioplasty and stenting in single center. Catheter Cardiovasc Interv. (2014) 83:1. 10.1002/ccd.25038 23729240

[13] Goyal M Menon BK van Zwam WH Dippel DW Mitchell PJ Demchuk AM. Endovascular thrombectomy after large-vessel ischaemic stroke: a meta-analysis of individual patient data from five randomised trials. Lancet. (2016) 387:1723–31. 10.1016/S0140-6736(16)00163-X 26898852

[14] Saver JL Goyal M van der Lugt A Menon BK Majoie CB Dippel DW. Time to treatment with endovascular thrombectomy and outcomes from ischemic stroke: a meta-analysis. JAMA. (2016) 316:1279–88. 10.1001/jama.2016.13647 27673305

[15] Alawieh A Vargas J Fargen KM Langley EF Starke RM De Leacy R. Impact of procedure time on outcomes of thrombectomy for stroke. J Am Coll Cardiol. (2019) 73:879–90. 10.1016/j.jacc.2018.11.052 30819354

[16] Nogueira RG Liebeskind DS Sung G Duckwiler G Smith WS. Predictors of good clinical outcomes, mortality, and successful revascularization in patients with acute ischemic stroke undergoing thrombectomy: pooled analysis of the Mechanical Embolus Removal in Cerebral Ischemia (MERCI) and Multi MERCI Trials. Stroke. (2009) 40:3777–83. 10.1161/STROKEAHA.109.561431 19875740

[17] Hao Y Yang D Wang H Zi W Zhang M Geng Y. Predictors for symptomatic intracranial hemorrhage after endovascular treatment of acute ischemic stroke. Stroke. (2017) 48:1203–9. 10.1161/STROKEAHA.116.016368 28373302

[18] Roman LS Menon BK Blasco J Hernandez-Perez M Davalos A Majoie C. Imaging features and safety and efficacy of endovascular stroke treatment: a meta-analysis of individual patient-level data. Lancet Neurol. (2018) 17:895–904. 10.1016/S1474-4422(18)30242-4 30264728

[19] Eker OF Buhlmann M Dargazanli C Kaesmacher J Mourand I Gralla J. Endovascular treatment of atherosclerotic tandem occlusions in anterior circulation stroke: technical aspects and complications compared to isolated intracranial occlusions. Front Neurol. (2018) 9:1046. 10.3389/fneur.2018.01046 30619028 PMC6300468

[20] Chartrain AG Awad AJ Mascitelli JR Shoirah H Oxley TJ Feng R. Novel and emerging technologies for endovascular thrombectomy. Neurosurg Focus. (2017) 42: E12. 10.3171/2017.1.FOCUS16518 28366058

[21] Leung V Sastry A Srivastava S Wilcock D Parrott A Nayak S. Mechanical thrombectomy in acute ischaemic stroke: a review of the different techniques. Clin Radiol. (2018) 73:428–38. 10.1016/j.crad.2017.10.022 29329730

[22] Puetz V Sylaja PN Coutts SB Hill MD Dzialowski I Mueller P. Extent of hypoattenuation on CT angiography source images predicts functional outcome in patients with basilar artery occlusion. Stroke. (2008) 39:2485–90. 10.1161/STROKEAHA.107.511162 18617663

[23] van der Hoeven EJ McVerry F Vos JA Algra A Puetz V Kappelle LJ. Collateral flow predicts outcome after basilar artery occlusion: the posterior circulation collateral score. Int J Stroke. (2016) 11:768–75. 10.1177/1747493016641951 27016515

