# Corrigendum: Learning Curve for Endovascular Treatment of Anterior Circulation Large Vessel Occlusion at a Single Center

**DOI:** 10.3389/fneur.2021.703055

**Published:** 2021-06-02

**Authors:** Qiankun Cai, Yuyou Zhu, Xianjun Huang, Lulu Xiao, Mengmeng Gu, Peng Wang, Chao Zhang, Jixing Chen, Wei Hu, Guoping Wang, Wen Sun

**Affiliations:** ^1^Department of Neurology, Second Affiliated Hospital, Fujian Medical University, Quanzhou, China; ^2^Department of Neurology, Stroke Center, The First Affiliated Hospital of University of Science and Technology of China, Hefei, China; ^3^Department of Neurology, Yijishan Hospital of Wannan Medical College, Wuhu, China; ^4^Department of Neurology, Jinling Hospital, Medical School of Nanjing University, Nanjing, China; ^5^Department of Neurology, Nanjing First Hospital, Nanjing Medical University, Nanjing, China; ^6^Department of Radiology, Stroke Center, The First Affiliated Hospital of University of Science and Technology of China, Hefei, China

**Keywords:** learning curve, endovascular treatment, large vessel occlusion, risk adjusted cumulative sum, stroke

In the original article, there was an error. We neglected to state that the authors Qiankun Cai and Yuyou Zhu contributed equally to this work.

The authors apologize for this error and state that this does not change the scientific conclusions of the article in any way. The original article has been updated.

