# Supplementary material for: Learning Curve for Endovascular Treatment of Anterior Circulation Large Vessel Occlusion at a Single Center
Source: Front Neurol. 2021 Jan 14;11:587409. doi: 10.3389/fneur.2020.587409 (PMC7840614; doi:10.3389/fneur.2020.587409)
Supplement: Supplementary file 1 [file Table_1.DOCX]

**SUPPLEMENTAL MATERIAL**

**Supplemental Table I. Outcome comparison across operators**

|  | **Operator A**  **N=61** | **Operator B**  **N=54** | **Operator C**  **N=56** | **Operator D**  **N=54** | **Operator E**  **N=58** | **Operator F**  **N=79** | **Operator G**  **N=72** | **P** |
| --- | --- | --- | --- | --- | --- | --- | --- | --- |
| SR | 44 (72.1) | 42 (77.8) | 47 (83.9) | 45 (83.3) | 47 (81.0) | 64 (81.0) | 59 (81.9) | 0.715 |
| FI | 31 (50.8) | 24 (44.4) | 22 (39.3) | 22 (40.7) | 29 (50.0) | 34 (43.0) | 31 (43.1) | 0.839 |
| 90-day mortality | 10 (16.4) | 12 (22.2) | 12 (21.4) | 11 (20.4) | 13 (22.4) | 22 (27.8) | 20 (27.8) | 0.705 |
| SICH | 6 (9.8) | 3 (5.6) | 4 (7.1) | 6 (11.1) | 6 (10.3) | 13 (16.5) | 13 (18.1) | 0.243 |
| PRT, min | 80 (46-116) | 68 (49-92) | 60 (44-118) | 68 (43-105) | 66 (44-102) | 85 (52-108) | 79 (50-102) | 0.340 |
| mRS score | 2 (1-5) | 3 (1-5) | 3 (1-5) | 3 (1-4) | 3 (1-5) | 3 (1-6) | 4 (1-6) | 0.898 |

Continuous variables are presented as the median (interquartile range), and categorical variables are presented as the frequency (%).

Abbreviations: SR, successful reperfusion; FI, functional independence; SICH, symptomatic intracerebral hemorrhage; PRT, puncture-to-reperfusion time; mRS, modified Rankin Scale.

**Supplemental Table II. Univariate analysis for functional independence, 90-day mortality and successful reperfusion.**

|  | **Functional independence** | | **P** | **90-day mortality** | | **P** | **Successful reperfusion** | | | **P** |
| --- | --- | --- | --- | --- | --- | --- | --- | --- | --- | --- |
|  | No (n=241) | Yes (n=193) |  | No (n=334) | Yes (n=100) |  | No (n=86) | Yes (n=348) | |  |
| Age, year | 70 (61-78) | 61 (53-70) | <0.001 | 65 (55-72) | 72 (64-79) | <0.001 | 70 (60-78) | 66 (56-73) | | 0.043 |
| Male | 149 (61.8) | 135 (69.9) | 0.077 | 225 (67.4) | 59 (59.0) | 0.123 | 52 (60.5) | 232 (64.1) | | 0.279 |
| NIHSS | 19 (16-22) | 15 (11-18) | <0.001 | 16 (13-20) | 19 (16-22) | <0.001 | 18 (15-21) | 17 (13-20) | | 0.657 |
| Hypertension | 160 (66.4) | 120 (62.2) | 0.362 | 210 (62.9) | 70 (70.0) | 0.191 | 57 (66.3) | 223 (64.1) | | 0.703 |
| DM | 58 (24.1) | 43 (22.3) | 0.662 | 80 (24.0) | 21 (21.0) | 0.540 | 19 (22.1) | 82 (23.6) | | 0.773 |
| Dyslipidemia | 61 (25.3) | 42 (21.8) | 0.388 | 73 (21.9) | 30 (30.0) | 0.093 | 19 (22.1) | 84 (24.1) | | 0.690 |
| Atrial fibrillation | 114 (47.3) | 55 (28.5) | <0.001 | 114 (34.1) | 55 (55.0) | <0.001 | 37 (43.0) | 132 (37.9) | | 0.386 |
| Current smoking | 66 (27.4) | 73 (37.8) | 0.021 | 116 (34.7) | 23 (23.0) | 0.027 | 19 (22.1) | 120 (34.5) | | 0.027 |
| Stroke cause |  |  | 0.066 |  |  | 0.059 |  |  | | 0.101 |
| cardioembolism | 122 (50.6) | 76 (39.3) |  | 142 (42.5) | 56 (56.0) |  | 33 (38.4) | 165 (47.4) | |  |
| LAA | 99 (41.1) | 95 (49.2) |  | 158 (47.3) | 36 (36.0) |  | 40 (46.5) | | 154 (44.3) |  |
| Other | 20 (8.3) | 22 (11.4) |  | 34 (10.2) | 8 (8.0) |  | 13 (15.1) | | 29 (8.3) |  |
| ASPECTS | 9 (7-10) | 9 (8-10) | <0.001 | 9 (8-10) | 9 (6-10) | 0.021 | 9 (7-10) | | 9 (8-10) | 0.020 |
| Occlusion site |  |  | 0.004 |  |  | 0.002 |  | |  | 0.007 |
| MCA M1 | 116 (48.1) | 118 (61.1) |  | 195 (58.4) | 39 (39.0) |  | 34 (39.5) | | 200 (57.5) |  |
| ICA | 103 (42.7) | 54 (28.0) |  | 108 (32.3) | 49 (49.0) |  | 45 (52.3) | | 112 (32.2) |  |
| MCA M2 | 17 (7.1) | 20 (10.4) |  | 28 (8.4) | 9 (9.0) |  | 6 (7.0) | | 31 (8.9) |  |
| ACA | 5 (2.1) | 1 (0.5) |  | 3 (0.9) | 3 (3.0) |  | 1 (1.2) | | 5 (1.4) |  |
| Tandem lesion | 46 (19.1) | 36 (18.7) | 0.909 | 64 (19.2) | 18 (18.0) | 0.795 | 29 (33.7) | | 53 (15.2) | <0.001 |
| Good collateral | 97 (40.2) | 135 (69.9) | <0.001 | 198 (59.3) | 34 (34.0) | <0.001 | 33 (38.4) | | 199 (57.2) | 0.002 |
| Left hemisphere | 137 (56.8) | 104 (53.9) | 0.537 | 180 (53.9) | 61 (61.0) | 0.279 | 50 (58.1) | | 191 (54.9) | 0.587 |
| OPT, min | 270  (225-358) | 297  (230-374) | 0.113 | 292  (230-371) | 268  (213-350) | 0.041 | 294  (230-362) | | 284  (222-364) | 0.125 |
| DPT, min | 112  (90-139) | 110  (86-141) | 0.952 | 113  (87-142) | 111  (90-126) | 0.337 | 118  (92-151) | | 110  (87-135) | 0.068 |
| Thrombolysis IV | 62 (25.7) | 66 (34.2) | 0.054 | 100 (29.9) | 28 (28.0) | 0.709 | 24 (27.9) | 104 (29.9) | | 0.719 |
| Retriever stent | 216 (89.6) | 161 (83.4) | 0.057 | 289 (86.5) | 88 (88.0) | 0.702 | 68 (79.1) | 309 (88.8) | | 0.017 |
| Angioplasty/stent | 10 (4.1) | 20 (10.4) | 0.011 | 25 (7.5) | 5 (5.0) | 0.390 | 7 (8.1) | 23 (6.6) | | 0.616 |
| Aspiration | 5 (2.1) | 12 (6.2) | 0.027 | 15 (4.5) | 2 (2.0) | 0.381 | 2 (2.3) | 15 (4.3) | | 0.543 |
| Local anesthesia | 223 (92.5) | 180 (93.3) | 0.768 | 313 (93.7) | 90 (90.0) | 0.206 | 80 (93.0) | 123 (92.8) | | 0.947 |
| Carotid stent | 11 (4.6) | 16 (8.3) | 0.110 | 23 (6.9) | 4 (4.0) | 0.295 | 7 (8.1) | 20 (5.7) | | 0.411 |
| Rescue therapy | 100 (41.5) | 87 (45.1) | 0.454 | 142 (42.5) | 45 (45.0) | 0.660 | 36 (41.9) | 151 (43.4) | | 0.797 |

Continuous variables are presented as the median (interquartile range), and categorical variables are presented as the frequency (%).

Univariate analysis was compared with or Mann-Whitney U test for continuous variables and Pearson’s chi-square or Fisher’s exact test for categorical variables.

Abbreviations: NIHSS, National Institutes of Health Stroke Scale; DM, diabetes mellitus; LAA, large artery atherosclerosis; ASPECTS, Alberta Stroke Program Early CT score; ICA, internal carotid artery; MCA, middle cerebral artery; ACA, anterior cerebral artery; OPT, onset-to-puncture time; DPT, door-to-puncture time; IV, intravenous.

**Supplemental Table III. Correlation analysis for puncture-to-reperfusion time.**

|  | **Puncture-to-reperfusion time** | ***P*** |
| --- | --- | --- |
|  | **Correlation coefficient** |  |
| Age, year | 0.032 | 0.511 |
| Sex (male vs female) | -0.053 | 0.276 |
| NIHSS | 0.125 | 0.010 |
| Hypertension (yes vs no) | 0.047 | 0.336 |
| Diabetes mellitus (yes vs no) | 0.024 | 0.619 |
| Dyslipidemia (yes vs no) | 0.011 | 0.825 |
| Atrial fibrillation (yes vs no) | -0.008 | 0.868 |
| Current smoking (yes vs no) | -0.091 | 0.061 |
| Cardioembolism (yes vs no) | -0.121 | 0.013 |
| LAA (yes vs no) | 0.065 | 0.180 |
| Other (yes vs no) | 0.096 | 0.047 |
| ASPECTS | 0.067 | 0.166 |
| MCA-M1 occlusion (yes vs no) | -0.150 | 0.002 |
| ICA occlusion (yes vs no) | 0.165 | 0.001 |
| MCA-M2 occlusion (yes vs no) | -0.040 | 0.408 |
| ACA occlusion (yes vs no) | 0.046 | 0.342 |
| Tandem lesion (yes vs no) | 0.225 | <0.001 |
| Collateral (good vs poor) | -0.129 | 0.008 |
| Affected hemisphere (right vs left) | -0.037 | 0.447 |
| Onset-to-puncture time | 0.044 | 0.363 |
| Door-to-puncture time | 0.041 | 0.400 |
| Intravenous thrombolysis (yes vs no) | 0.101 | 0.038 |
| Retriever stent (yes vs no) | -0.017 | 0.722 |
| Angioplasty/stent (yes vs no) | 0.068 | 0.161 |
| Aspiration (yes vs no) | -0.130 | 0.007 |
| Anesthesia (local vs general) | 0.001 | 0.979 |
| Carotid stent (yes vs no) | 0.126 | 0.009 |
| Rescue therapy (yes vs no) | 0.162 | <0.001 |

Abbreviations: NIHSS, National Institutes of Health Stroke Scale; LAA, large artery atherosclerosis; ASPECTS, Alberta Stroke Program Early CT score; ICA, internal carotid artery; MCA, middle cerebral artery; ACA, anterior cerebral artery.

**Supplemental Table IV. Variables associated with functional independence, 90-day mortality and puncture-to-reperfusion time and successful reperfusion in multivariable backward regression models**

| **Variables** | **Β** | **OR** | **95% CI** | **P** |
| --- | --- | --- | --- | --- |
| **Functional independence**^†^ |  |  |  |  |
| Case number (per increase 10) | 0.198 | 1.219 | 1.079 to 1.383 | <0.001 |
| Age (per increase 1) | -0.049 | 0.952 | 0.934 to 0.971 | <0.001 |
| NIHSS (per increase 1) | -0.134 | 0.875 | 0.835 to 0.916 | <0.001 |
| Intravenous Thrombolysis | 0.852 | 2.343 | 1.417 to 3.874 | <0.001 |
| Collateral (good vs poor) | 0.983 | 2.673 | 1.676 to 4.262 | <0.001 |
| **90-day mortality**^†^ |  |  |  |  |
| Case number (per increase 10) | -0.166 | 0.847 | 0.738 to 0.968 | 0.016 |
| Age (per increase 1) | 0.056 | 1.057 | 1.033 to 1.082 | <0.001 |
| MCA-M1 occlusion (yes vs no) | -0.651 | 0.522 | 0.319 to 0.853 | 0.010 |
| ASPECTS (per increase 1) | -0.136 | 0.873 | 0.764 to 0.997 | 0.045 |
| Collateral (good vs poor) | -0.766 | 0.465 | 0.281 to 0.769 | 0.003 |
| **Puncture-to-reperfusion time**^‡^ |  |  |  |  |
| Case number (per increase 10) | -8.087 |  | -9.991 to -6.184 | <0.001 |
| Rescue therapy (yes vs no) | 14.302 |  | 7.088 to 21.517 | <0.001 |
| Collateral (good vs poor) | -8.583 |  | -15.810 to -1.356 | 0.020 |
| Tandem lesion (yes vs no) | 26.518 |  | 17.275 to 35.762 | <0.001 |
| Aspiration (yes vs no) | -21.170 |  | -39.432 to -2.908 | <0.001 |
| **Successful reperfusion**^†^ | | | | |
| Case number (per increase 10) | 0.440 | 1.553 | 1.332 to 1.830 | <0.001 |
| Tandem lesion (yes vs no) | -1.140 | 0.320 | 0.177 to 0.578 | <0.001 |
| Current Smoking (yes vs no) | 0.741 | 2.098 | 1.144 to 3.848 | 0.017 |
| Collateral (good vs poor) | 0.724 | 2.062 | 1.224 to 3.472 | 0.007 |

†Logistic backward regression models were performed by adjusting for covariates with *P*-value<0.1 in univariate analysis.

‡There were 8 patients missing data. Linear backward regression model was performed adjusting covariates with *P*-value<0.1 in univariate correlation analysis.

Abbreviations: NIHSS, National Institutes of Health Stroke Scale; MCA, middle cerebral artery; ASPECTS, Alberta Stroke Program Early CT score.

**Supplemental Table V. Univariate analysis and multivariable logistic regression for symptomatic intracerebral hemorrhage**

|  | **SICH** | |  | **Logistic model*** |  |
| --- | --- | --- | --- | --- | --- |
|  | **No**  **(n=383)** | **Yes**  **(n=51)** | **P** | **OR 95% CI** | **P** |
| Age, year | 67(57-74) | 66(57-74) | 0.927 |  |  |
| Male | 247(64.5) | 37(72.5) | 0.256 |  |  |
| NIHSS | 17(13-21) | 18(16-21) | 0.012 |  |  |
| Hypertension | 252(63.8) | 28(54.9) | 0.127 |  |  |
| DM | 87(22.7) | 14(27.5) | 0.452 |  |  |
| Dyslipidemia | 88(23.0) | 15(29.4) | 0.310 |  |  |
| Atrial fibrillation | 147(38.4) | 22(43.1) | 0.513 |  |  |
| Current smoking | 129(33.7) | 10(19.6) | 0.043 | 0.465 0.224-0.963 | 0.039 |
| Stroke cause |  |  | 0.229 |  |  |
| Cardioembolism | 169(44.1) | 29(56.9) |  |  |  |
| LAA | 176(46.5) | 18(35.3) |  |  |  |
| Other | 38(9.9) | 4(7.8) |  |  |  |
| ASPECT | 9(8-10) | 9(7-10) | 0.386 |  |  |
| Occlusion site |  |  | 0.095 |  |  |
| MCA M1 | 214(55.9) | 20(39.2) |  |  |  |
| ICA | 131(34.2) | 26(51.0) |  |  |  |
| MCA M2 | 32(8.4) | 5(9.8) |  |  |  |
| ACA | 6(1.6) | 0(0) |  |  |  |
| Tandem lesion | 72(18.8) | 10(19.6) | 0.890 |  |  |
| Good collateral | 214(55.9) | 18(35.3) | 0.006 | 0.421 0.228-0.776 | 0.006 |
| Left hemisphere | 210(54.8) | 31(60.8) | 0.422 |  |  |
| OPT, min | 290  (228-364) | 270  (220-355) | 0.463 |  |  |
| DPT, min | 113  (90-140) | 105  (82-128) | 0.172 |  |  |
| Thrombolysis IV | 109(28.5) | 19(37.3) | 0.196 |  |  |
| Retriever stent | 331(86.4) | 46(90.2) | 0.454 |  |  |
| Angioplasty/stent | 28(7.3) | 2 (3.9) | 0.558 |  |  |
| Aspiration | 16(4.2) | 1 (2.0) | 0.706 |  |  |
| Local anesthesia | 358(93.5) | 45(88.2) | 0.240 |  |  |
| Carotid stent | 26(6.8) | 1(2.0) | 0.348 |  |  |
| Rescue therapy | 166(43.3) | 21(41.2) | 0.769 |  |  |

Continuous variables are presented as the median (interquartile range), and categorical variables are presented as the frequency (%).

Univariate analysis was performed with Mann-Whitney U test for continuous variables and Pearson’s chi-square or Fisher’s exact test for categorical variables.

*Multivariable logistic backward regression-adjusted covariates with *P*-value<0.1 in the univariate analysis.

Abbreviations: NIHSS, National Institutes of Health Stroke Scale; DM, diabetes mellitus; LAA, large artery atherosclerosis; ASPECT, Alberta Stroke Program Early CT; ICA, internal carotid artery; MCA, middle cerebral artery; ACA, anterior cerebral artery; OPT, onset-to-puncture time; DPT, door-to-puncture time; IV, intravenous.

**Risk-adjusted cumulative sum (RA-CUSUM) chart**

This method is an extension of the original CUSUM analysis, which plots the difference between cumulative expected and observed events. The formula is as follows: C_n_= C_n-1_+ (P_n_ - X_n_), where C_0_= 0; n= the number of cases (i.e., the operators’ 1^st^, 2^nd^, 3^rd^ … or n^th^ case); X_n_= the observed outcome of the n^th^ case for binary outcome; X= 0 for a case without successful reperfusion; X= 1 for a case with successful reperfusion; and P_n_= the expected probability of successful reperfusion itself, regardless of operator experience, for the n^th^ case (calculated by the logistic regression model for successful reperfusion, see **Supplemental Table VI**). In this study, the RA-CUSUM chart assessed the learning curve of EVT for several operators; therefore, C_n_= the cumulative sum of the expected probability of successful reperfusion for all operators during their 1^st^, 2^nd^, 3^rd^… and n^th^ case minus the observed successful reperfusion. The chart was plotted with the X-axis representing “n” and the Y-axis representing “C_n_”. For each case, the expected probability of successful reperfusion was calculated by logistic regression, which in turn determined the gradient of graph deflection. For example, the graph ascended by an amount equal to P_n_ when the n^th^ case did not achieve successful reperfusion (punishment) or descended by an amount equal to (1-P_n_) when successful reperfusion was achieved (award). Thus, if an easy case (a high value of P) demonstrated successful reperfusion, the graph only descended by a small value equal to (1-P), i.e., the case was not unduly rewarded and vice versa. Therefore, the RA-CUSUM chart was adjusted for case mixing.

For the n^th^ case in this study, the predicted probability of successful reperfusion was calculated based on results in **Supplemental Table VI** as follows:

**P=1/(1+exp (1.141*tandem lesion-0.802*Smoke-0.780*Collateral-1.072)).**

**Supplemental Table VI. Variables associated with successful reperfusion in a multivariable logistic backward regression model for RA-CUSUM.**

| **Variables** | **Β** | **OR** | **95% CI** | **P** |
| --- | --- | --- | --- | --- |
| **Successful reperfusion** | | | | |
| Tandem lesion (yes vs no) | -1.141 | 0.320 | 0.183 to 0.558 | <0.001 |
| Current smoking (yes vs no) | 0.802 | 2.231 | 1.245 to 3.997 | 0.007 |
| Collateral (good vs bad) | 0.780 | 2.181 | 1.326 to 3.587 | 0.002 |
| Intercept | 1.072 |  |  |  |

The predicted probability of successful reperfusion itself, regardless of operator experience, was calculated by another backward model adjusting only for covariates with *P*-value<0.1 in the univariate analysis for the RA-CUSUM chart.
